# Psychometric Properties of the World Health Organization’s Quality of Life (WHOQOL-BREF) Questionnaire in Medical Students

**DOI:** 10.3390/medicina55120772

**Published:** 2019-12-04

**Authors:** Irena Ilić, Sandra Šipetić-Grujičić, Jovan Grujičić, Ivana Živanović Mačužić, Sanja Kocić, Milena Ilić

**Affiliations:** 1 Faculty of Medicine, University of Belgrade, 11000 Belgrade, Serbia; 2 Institute of Epidemiology, Faculty of Medicine, University of Belgrade, 11000 Belgrade, Serbia; sandra.grujicic2014@gmail.com; 3 Department of Biochemistry, Ave Maria University, Ave Maria, FL 34142, USA; ssipetic@yahoo.com; 4 Department of Anatomy, Faculty of Medical Sciences, University of Kragujevac, 34000 Kragujevac, Serbia; ivanaanatom@yahoo.com; 5 Department of Social Medicine, Faculty of Medical Sciences, University of Kragujevac, 34000 Kragujevac, Serbia; kocicsanja@yahoo.com; 6 Department of Epidemiology, Faculty of Medical Sciences, University of Kragujevac, 34000 Kragujevac, Serbia; drmilenailic@yahoo.com

**Keywords:** quality of life, WHOQOL-BREF, medical students, reliability, validity

## Abstract

*Background and Objectives:* Studies on the effects of studying on a medical student’s quality of life are sparse. The World Health Organization Quality of Life (WHOQOL-BREF) questionnaire is a widely used scale that enables the assessment and international comparisons of the quality of life. The aim of this study was to evaluate psychometric properties of the WHOQOL-BREF questionnaire among Serbian medical students. *Material and Methods:* We conducted a cross-sectional study that involved 760 medical students at a state medical faculty at the University of Kragujevac, Serbia. The reliability of the WHOQOL-BREF was evaluated using Cronbach’s alpha coefficient and test–retest analysis, and the validity was examined using principal component analysis, with Promax rotation method. *Results:* Cronbach’s alpha coefficient for the whole WHOQOL-BREF scale was 0.896. Internal reliability for all domains was above 0.70, except for the domain “Social Relationships” (0.533). The test–retest reliability for all domains was significant at *p* < 0.01 level, showing good stability of the scale. Principal component analysis with Promax rotation method indicated four main components that explained 49.5% of variance. *Conclusion:* The Serbian version of the WHOQOL-BREF scale showed satisfactory psychometric properties that facilitate estimation of the quality of life of medical students.

## 1. Introduction

The study of medicine is a long-lasting and strenuous process during which the quality of life of medical students can worsen [1,2]. Despite the numerous studies suggesting that medical students are experiencing anxiety, depression, and burnout, there are not many studies that deal with the assessment of the quality of life in this student population [3,4].

According to the World Health Organization, quality of life is defined as “individuals’ perceptions of their position in life in the context of the culture and value systems in which they live and in relation to their goals, expectations, standards and concerns” [5]. One of the measurement instruments which provides a reliable assessment of quality of life is the WHOQOL-BREF, questionnaire of the World Health Organization’s Quality of Life—Biomedical Research and Education Facility [6,7]. The WHOQOL-BREF is a questionnaire widely used as a generic measure of quality of life [7,8,9,10]. The WHOQOL-BREF contains 26 items which evaluate the four dimensions of quality of life (“Physical Health”, “Psychological Health”, “Social Relationships”, “Environment”), and two items that are examined separately (an individual’s “overall perception of quality of life” and an individual’s “overall perception of health”) [5]. The WHOQOL-BREF has been translated into several languages and validation studies were conducted in different countries over the past years [7,8,9]. Numerous studies in heterogeneous samples (both in the general population and in those suffering from different diseases) showed satisfactory psychometric properties of the WHOQOL-BREF questionnaire [6,7,8,9,10], but only a few studies were conducted in a sample of medical students [11,12]. The collaborative assessment of the WHOQOL-BREF scale in 15 cultural settings over several years confirmed good internal consistency (Cronbach’s alpha coefficients for the domain scores ranged from 0.66 to 0.84) [6]. Also, assessment of the WHOQOL-BREF in some countries (Brazil, Iran, Spain) confirmed good internal consistency (Cronbach’s alpha coefficient > 0.7) [9,13,14]. Most of the validation studies revealed a four-factor structure of the WHOQOL-BREF scale [6,11,15]. Similar to studies in Australia [11] and China [12], one recent study showed that the WHOQOL-BREF tool is valid (four domains) and reliable (the Cronbach’s α coefficient was over 0.7 for the questionnaire as a whole, and for all domains) for assessing quality of life among Saudi medical students [16]. In one validation study in Turkey [17], both structure and convergent validity for the WHOQOL-BREF scale were satisfactory. However, in a recent validation study [18], the Cattelle’s Scree plot, Horn’s parallel analysis and the confirmatory factor analysis revealed a two-factor model as the best model for the adapted WHOQOL-BREF tool in a large sample of adolescents in Nigeria.

Although a few studies have assessed the validity and reliability of the WHOQOL-BREF questionnaire in Serbia [19], a comprehensive evaluation of its psychometric properties is lacking. The aim of this study was to evaluate psychometric properties of the WHOQOL-BREF quality of life questionnaire in Serbian medical students.

## 2. Materials and Methods

### 2.1. Study Design and Setting

A cross-sectional study design was used in this research. The research was conducted during the first week of classes of the second semester of the academic year 2013–2014, at the Faculty of Medical Sciences, University of Kragujevac (Serbia). The Faculty of Medical Sciences within the University of Kragujevac organizes the program of the academic integrated studies of medicine through blocks (semesters), and it consists of 12 blocks with 50 courses (35 courses are obligatory and 15 are elective). Medical studies last six years. All courses last one semester. The teaching process is realized through lectures, laboratory sessions, other forms of lecturing, and professional practice. In past years, a relatively small number of students were enrolled, in order to fulfill the set standards of the quality of program. Self-reported questionnaires were distributed to all medical students who attended classes. The survey was performed in classrooms, according to previous agreement with the professors.

### 2.2. Study Sample

The study population consisted of all medical students enrolled in the academic integrated studies at the Faculty of Medical Sciences Kragujevac during the academic study year 2013/2014.

The criteria for the involvement of students in this study were age (18 years or older), voluntary informed written consent to participate, completely filling all items on the WHOQOL-BREF and additionally, for retesting only, participation in both the first and repeated testing.

### 2.3. Data Collection and Study Timepoints

After the approval of Ethical Committee, the research began in such a way that it did not interfere with teaching at the faculty. Students received written information on the research, which explained the purpose and method of conducting the research, the expected contributions of the study, and the ethical issues. Additionally, the information indicated that research is completely anonymous and voluntary, and that the data obtained will be used solely for research purposes and analyzed at group level. All the time, students had the opportunity to ask questions if something was unclear about the research. Before completing the questionnaire, the participants gave written consent to participate in the research. Students did not receive any compensation, nor were they motivated in any other way to participate in the research. At any moment, respondents could leave the research without any consequences. Completing the questionnaire took about 15 (±5) minutes. Questionnaires that were not fully completed were not included in the analysis.

The research was conducted in two ‘study time points’, as test and retest surveys. The retest was conducted during the lecture in the same course and included the same medical students (freshmen only) which fulfilled the initial test. ‘Study time-point 1’ represented the time when participants entered the study, provided voluntary informed written consent, and filled out the ‘initial’ survey (WHOQOL-BREF—testing). The next study time-point (‘time-point 2’) represented the moment when the participants completed the questionnaire for the second time (WHOQOL-BREF—retesting), approximately 3 weeks after the initial testing. The initial test and retest were conducted under the same conditions, in the presence of a medical doctor (authors M.I. and I.I.) who were available to address any difficulties in the student’s understanding of certain issues.

### 2.4. Instruments

Epidemiological questionnaire was designed for the purpose of examining some sociodemographic (such as age and gender) and lifestyle/health facts (such as smoking, alcohol use, sports and recreation activity, and personal medical history).

WHOQOL-BREF scale contains a total of 26 items: items 3–26 represent four domains (“Physical Health”—7 items (Pain and discomfort; Dependence on medicinal substances and medical aids; Energy and fatigue; Mobility; Sleep; Activities of daily living; Work capacity),“Psychological Health”—6 items (Positive feelings; Spirituality/personal beliefs; Thinking, learning, memory and concentration; Bodily image and appearance; Self-esteem; Negative feelings), “Social Relationships”—3 items (Personal relationships; Sexual activity; Social support), “Environment”—8 items (Freedom, physical safety, and Security; Physical environment (pollution/noise/traffic/climate); Financial resources; Opportunities for acquiring new information and skills; Participation in and opportunities for recreation/leisure activities; Home environment; Health and social care: accessibility and quality; Transport)) and, two items (1 and 2) that are examined separately and refer to an individual’s “Overall perception of quality of life” and an individual’s “Overall perception of health” [6,7]. Students were asked how they self-assessed their quality of life, their health status and other areas of their lives over the last two weeks. The answers for each item are given on a 1–5 Likert-type scale, where 1 denotes the least, and 5 is the highest agreement with a particular claim. Items 3, 4, and 26 are negatively phrased and reversed during analysis. The result is calculated on four domains of quality of life and on two separate items that measure overall perception of quality of life and overall perception of health. The mean score of items within each domain is used to calculate the domain score. Results on domains represent the sum of results of items. A higher sum of points represents a higher quality of life on a single domain.

The translation followed an internationally accepted, back-translation methodology [20]. Firstly, the WHOQOL-BREF was translated from English to Serbian (“forward translation”) by two independent, professional translators. After, translation of the Serbian version of WHOQOL-BREF questionnaire to English language (“backward translation”) was conducted by two translators, one an expert in quality of life and one an epidemiologist, with discussion on ambiguity regarding certain items. Then, the Serbian version of the WHOQOL-BREF questionnaire was presented to five medical students, in order to check the understanding of translated items. Тhereafter, any comments which were made during the testing were considered by the study authors. Finally, the final Serbian version of the WHOQOL-BREF questionnaire was tested on a sample of medical students.

### 2.5. Statistical Analysis

In the descriptive statistics, categorical variables (presented as percentages) and continuous variables (presented as mean ± standard deviation) were calculated.

The classical test theory was used in data analysis. Reliability analyses were carried out using internal consistency reliability and test–retest reliability for each dimension. Internal consistency (as the extent to which each domain forms a reliable scale) was assessed using standardized Cronbach’s coefficient alpha. Values of the Cronbach’s alpha coefficient ≥ 0.7 are acceptable, while values ≥ 0.8 are preferred. Also, the correlation of items with a simple linear combination of all other items (Item–Test Score Correlation) was calculated. Test–retest reliability was assessed using Pearson’s *r* correlations. Also, Pearson’s correlation coefficient was used to investigate the association between scores of the WHOQOL-BREF domains and perception of overall quality of life and perception of overall general health. To evaluate construct validity, an exploratory factor analysis was performed. Kaiser–Meyer–Olkin Measure of Sampling Adequacy and Bartlett’s Test of Sphericity were used to assess the suitability of data for structure evaluation. Then, an exploratory factor analysis (principal component analysis as the extraction method, and Promax with Kaiser Normalization as the rotation method) was conducted. A factor was considered as important if its eigenvalue exceeded 1.0. The communality represents the percentage of variance of the tool item accounted for all factors. In order to strengthen the argument for the number of factors, parallel analysis (based on random data generation, by using the Monte Carlo simulation technique) was conducted. A *p*-value of < 0.05 was considered as statistically significant for all tests. All statistical analyses were conducted using the SPSS Software (version 20, Chicago, IL, USA).

### 2.6. Ethical Considerations

This study is part of a research approved by the Ethics Committee of the Faculty of Medical Sciences, University of Kragujevac (protocol: 01-1176, approved on 7th of February, 2014). All participants provided informed written voluntary consent before participation.

## 3. Results

A total of 760 medical students participated in the study (participation rate: 760/836 = 90.9%). Participation rate in the retesting, that included freshmen only, was 96.9% (93/96). Absence from the classes was the reason why some medical students are not included in the study, while the reasons for not accepting participation were a lack of interest or time. Analysis comprised only fully completed questionnaires.

The mean age of the participants was 23.7 ± 2.7 years (range 19–36 years) and 64.6% were female (Table 1). Medical students spent, on average, more than 4 h a day attending lectures and almost 5 h studying. Nearly 37% of the medical students had sport activities, while 76.4% participated in recreational physical activity. About one third of the medical students were smokers, while nearly two-thirds reported alcohol consumption. Personal history of chronic diseases was reported by 6.1% of participants, and they most often listed asthma, chronic sinusitis, migraine, arterial hypertension, and/or anemia.

The Serbian version of WHOQOL-BREF showed that the average items score varied from 3.13 to 4.77 (Table 2). Cronbach’s alpha coefficient for the whole WHOQOL-BREF scale (items 3–26) was 0.896. Internal reliability coefficients for the domains of the WHOQOL-BREF scale were above 0.70, except for domain “Social Relationships” (0.533). The intra-class correlation coefficients for the four components were (between 0.491 and 0.769) significant (*p* < 0.001).

The test–retest reliability correlation coefficients were significant at the 0.01 level for all domains: “Physical Health”—0.665, “Psychological Health”—0.724, “Social Relationships”—0.725, and “Environment”—0.602 (Table 3).

The scores of all of the WHOQOL-BREF domains correlated strongly with the scores of perception of overall quality of life and perception of overall general health (Table 4).

Results showed the Kaiser–Meyer–Olkin Measure to be 0.913 and the Bartlett’s Test of Sphericity to be chi-square = 5407.100 and *p* < 0.001, which indicated that the data in this study were suitable for factor analysis (Table 5). The factor analysis model revealed four factors with eigenvalue greater than 1, explaining 49.5% of cumulative variance. None of the items in the Serbian version of the WHOQOL-BREF scale corresponded fully to the original. The factors “Psychological Health” and “Social Relationships” could not be reproduced at all: namely, all of these items had the highest loading in the same component, and were jointed with questions 18 and 8 (which in the original version of the questionnaire were part of the domains “Physical Health” and “Environment”, respectively). Also, the items of other two domains (i.e., “Physical Health” and “Environment”) showed higher loadings on more than one factors. Additionally, the Parallel Analysis indicates that four components should be retained for the WHOQOL-BREF.

## 4. Discussion

The Serbian version of the WHOQOL-BREF questionnaire had satisfactory reliability and validity for assessing quality of life of medical students.

The psychometric properties of the WHOQOL-BREF scale were evaluated by numerous studies in the general population [7,18] and in different clinical populations [8,9], but very rarely in medical students [11,12]. In the available literature, we were not able to find data on the validation of the questionnaire WHOQOL-BREF among the Serbian population, except for the quality of life assessment among the persons who live in one retirement home in Novi Sad: this study showed that reliability was high for all four domains (Cronbach’s alpha ranged from 0.68 for “Social Relationships” to 0.79 for “Physical Health”) [19]. In our study, internal reliability for the WHOQOL-BREF domains was above 0.70, except for the domain “Social Relationships” (0.533). Similar, the international study (WHOQOL-BREF field trial) revealed that the internal consistency shown by Cronbach’s alpha for the domain “Social Relationships” in total was 0.68 [7]. The subscale “Social Relationships” included only three items: personal relationships, sexual activity, and social support. The reason for low internal reliability of this subscale most certainly could be the small number of items (3) [21]. Of course, a low value of alpha could be due to the cultural link between quality of life and confidentiality issue of questions about personal life in our population. In addition, a financial gap between students could have made it more difficult for them to attend normal social activities. Besides, the mean inter-item correlation for the items was 0.276 with strong intra-class correlation for the four components (*p* < 0.001), which suggested the optimal range of correlations between items [22]. Furthermore, some differences between studies have occurred most likely due to a large difference in age and gender between tested population groups or variations in understanding of questionnaires depending on literacy level, comorbidity, etc.

Good reliability (the Cronbach’s alpha was >0.7) of the WHOQOL-BREF scale has been determined in medical students in New Zealand [11], China [12], and Saudi Arabia [23]. Compared to that, internal consistencies of the WHOQOL-BREF scale were somewhat low in a sample of dental students in Saudi Arabia (“Psychological Health” domain showed unsatisfactory reliability having Cronbach’s Alpha of 0.36) [24], while reliability was low in the social domain in preclinical medical students in Iran (Cronbach’s alpha was 0.62 for “Social Relationships” domain) [25] and among Pakistani Urdu-speaking population aged 18 years and above (Cronbach’s alpha was 0.56 for “Social Relationships” domain) [13]. However, a study by Andre and coauthors [26] showed that reliability and structure validity of WHOQOL-BREF among students at one U.S. dental school were consistent with results in western students’ samples. Also, in the assessment of the quality of life among Chinese urologists [27], the overall Cronbach’s α coefficient of the instrument was 0.825, indicating the questionnaire was of good quality. Additionally, the test–retest reliability findings of our study which qualify WHOQOL-BREF as an excellent tool are consistent with previous studies [13,28].

Our study showed presence of four main factors with an eigenvalue larger than 1, explaining 49.5% of variance. In a study conducted in China, the results of factor analysis indicated that the four domains had the cumulative contribution rate of 69.3% [12]. In the international study (WHOQOL-BREF field trial), analysis of the total population data showed four factors that explained 53% of the variance, while center-specific analyses showed that most sites had four to six eigenvalues greater than 1.0 which explained 50–81% of variance [7].

Some differences in the psychometric characteristics of WHOQOL-BREF between different groups of medical students could be explained by differences in socio-demographic characteristics (age, study funding), cultural differences, differences in applied methodology, psychological support, and curriculum differences between samples of medical students.

### Strength and Limitations of the Study

A strength of this validation study was the assessment of reliability in two forms (test–retest reliability and internal consistency reliability) and its sample size (the participation rate of students in our study was satisfactory—90.9%). However, there were certain limitations in this study. Firstly, the use of self-reported data always is an important limitation. Second, our validity evaluation of the instrument was limited to the tests of exploratory construct validity. Furthermore, retesting was conducted among freshmen only, but they all spent more than six months at the university. Finally, questions about the sample size and the longitudinal design of the research always exist.

## 5. Conclusions

The Serbian version of the WHOQOL-BREF questionnaire has good psychometric properties and facilitates a satisfactory assessment of the quality of life in medical students. A better understanding of the quality of life may facilitate improvements in learning in medical students. Identification of good validity and reliability of the WHOQOL-BREF questionnaire among Serbian medical students may help to better understanding individuals across different types of cross-cultural research.

## Figures and Tables

**Table 1:** Descriptive statistics for the sample of medical students in the study

Variable	Number (760)	%
Female	491	64.6
Male	269	35.4
Age (year, mean ± SD)	23.7 ± 2.7	
Lectures (h/day, mean ± SD)	4.2 ± 1.5	
Studying (h/day, mean ± SD)	4.9 ± 2.2	
Sleeping	7.1 ± 1.3	
Sports	285	37.5
Recreation	581	76.4
Smoking	243	32.0
Alcohol use	455	59.9
Chronic diseases in personal history	46	6.1

**Table 2:** Internal consistency of the WHOQOL-BREF questionnaire in Serbian medical students

WHOQOL-BREF	Mean	Standard Deviation	Item-Test Score Correlation	Cronbach’s Coefficient	Cronbach’s α if Item Deleted	Intra-Class Correlation Coefficient
Perception of quality of life	3.87	0.794	NA	NA	NA	NA
Perception of health	3.92	0.853	NA	NA	NA	NA
Physical Health				0.744		0.733
Item 3	4.21	0.894	0.357		0.725	(*p* < 0.001)
Item 4	4.77	0.577	0.339		0.724	
Tem 10	3.88	0.768	0.624		0.661	
Item 15	4.11	0.943	0.389		0.719	
Item 16	3.51	0.991	0.413		0.714	
Item 17	3.97	0.733	0.655		0.657	
Item 18	4.16	0.746	0.426		0.706	
Psychological Health				0.777		0.769
Item 5	3.87	0.849	0.555		0.724	(*p* < 0.001)
Item 6	4.56	0.742	0.594		0.716	
Item 7	3.78	0.758	0.464		0.747	
Item 11	4.44	0.751	0.459		0.748	
Item 19	4.27	0.735	0.645		0.703	
Item 26	3.63	0.958	0.407		0.770	
Social Relationships				0.533		0.491
Item 20	4.35	0.646	0.411		0.294	(*p* < 0.001)
Item 21	3.92	1.107	0.289		0.502	
Item 22	4.30	0.786	0.293		0.419	
Environment						
Item 8	4.05	0.834	0.471	0.776	0.745	0.769
Item 9	3.13	1.065	0.408		0.758	(*p* < 0.001)
Item 12	3.70	0.929	0.536		0.733	
Item 13	4.37	0.713	0.430		0.753	
Item 14	3.58	0.953	0.516		0.736	
Item 23	4.26	0.770	0.514		0.739	
Item 24	3.28	1.037	0.444		0.750	
Item 25	3.47	1.079	0.481		0.744	

**Table 3:** Test–retest reliability of the WHOQOL-BREF questionnaire domains in Serbian medical students.

WHOQOL-BREF Domains	Physical Health—Repeated	Psychological Health—Repeated	Social Relationships—Repeated	Environment—Repeated
Physical Health	0.665 **	0.413 **	0.338 **	0.261 *
Psychological Health	0.516 **	0.724 **	0.445 **	0.322 **
Social Relationships	0.246 **	0.394 **	0.725 **	0.203
Environment	0.440 **	0.463 **	0.380 **	0.602 **

* *p* < 0.05; ** *p* < 0.01.

**Table 4:** Validity of the WHOQOL-BREF domains: correlation coefficients between scores of domains and perception of overall quality of life and perception of overall general health

WHOQOL-BREF Domains	Perception of Overall Quality of Life	Perception of Overall General Health
Physical Health	0.500 *	0.559 *
Psychological Health	0.574 *	0.450 *
Social Relationships	0.351 *	0.205 *
Environment	0.533 *	0.386 *

* *p* < 0.01.

**Table 5:** Principal component analysis with Promax rotation method for the WHOQOL-BREF scale items.

WHOQOL-BREF Scale Items	Correlation Coefficients of Variables and Factors *				Communalities
	Component				
	1	2	3	4	
Physical Health					
Item 3				0.737	0.586
Item 4				0.726	0.532
Tem 10	0.591	0.716		0.514	0.636
Item 15	0.432	0.434			0.260
Item 16		0.713			0.547
Item 17	0.564	0.723		0.423	0.593
Item 18	0.719	0.493			0.559
Psychological Health					
Item 5	0.677	0.540			0.508
Item 6	0.709				0.520
Item 7	0.531	0.530			0.380
Item 11	0.636				0.416
Item 19	0.830	0.401			0.693
Item 26	0.458	0.452		0.449	0.367
Social Relationships					
Item 20	0.739				0.563
Item 21	0.499	0.430			0.394
Item 22	0.524		0.436		0.400
Environment					
Item 8	0.622	0.482	0.428	0.417	0.481
Item 9		0.539			0.327
Item 12			0.758		0.578
Item 13			0.668		0.524
Item 14		0.542	0.567		0.465
Item 23			0.739		0.553
Item 24		0.533	0.525		0.472
Item 25		0.516	0.563		0.529
Variance explained (%)	30.7	7.2	6.5	5.1	
Cumulative variance (%)	30.7	37.9	44.4	49.5	
Kaiser–Meyer–Olkin Measure	0.913				
Bartlett’s Test of Sphericity	Chi-Square = 5407.100; df = 276; *p* < 0.001				

* Only factor weights greater than 0.4 are shown.
